# Unconsciously Triggered Conflict Adaptation

**DOI:** 10.1371/journal.pone.0011508

**Published:** 2010-07-09

**Authors:** Simon van Gaal, Victor A. F. Lamme, K. Richard Ridderinkhof

**Affiliations:** 1 Cognitive Neuroscience Group, Department of Psychology, University of Amsterdam, Amsterdam, The Netherlands; 2 Department of Psychology, Amsterdam Center for the Study of Adaptive Control in Brain and Behavior (Acacia), University of Amsterdam, Amsterdam, The Netherlands; 3 Cognitive Science Center, University of Amsterdam, Amsterdam, The Netherlands; Ecole Polytechnique Federale de Lausanne, Switzerland

## Abstract

In conflict tasks such as the Stroop, the Eriksen flanker or the Simon task, it is generally observed that the detection of conflict in the current trial reduces the impact of conflicting information in the subsequent trial; a phenomenon termed conflict adaptation. This higher-order cognitive control function has been assumed to be restricted to cases where conflict is experienced consciously. In the present experiment we manipulated the awareness of conflict-inducing stimuli in a metacontrast masking paradigm to directly test this assumption. Conflicting response tendencies were elicited either consciously (through primes that were weakly masked) or unconsciously (strongly masked primes). We demonstrate trial-by-trial conflict adaptation effects after conscious as well as unconscious conflict, which could not be explained by direct stimulus/response repetitions. These findings show that unconscious information can have a longer-lasting influence on our behavior than previously thought and further stretch the functional boundaries of unconscious cognition.

## Introduction

Masked priming studies have revealed a plethora of effects of masked stimuli on behavior and brain activity, highlighting an important role of unconscious information in guiding day-to-day behavior [1], [2], [3]. Masked priming studies also showed that the effects of unconscious stimuli on behavior and brain activity are of a fleeting form. Behavioral priming effects are generally absent when the interval between the masked prime and the target is longer than ∼500 ms [4], [5], [6] and neuroimaging studies showed a rapid decay of unconsciously triggered neural activations [7], often being absent in prefrontal cortex (PFC) [but see 8], [9], [10]. These results suggest that bridging information across time cannot occur if subjects are not aware of a stimulus [11]. On the other hand, conscious information can be held active for long periods of time and stored in working memory. The combination of these results suggests that conscious information can be used strategically to plan, guide and control future behaviors, whereas unconscious information cannot.

This phenomenon was elegantly illustrated by Kunde [12]. In his experiment, participants were required to perform a speeded two-choice response to a target arrow that was preceded by a smaller arrow, the so-called prime. Because the prime fitted within the contour of the target, the target functioned as a (metacontrast) mask [13], [14]. Therefore, participants were not aware of the prime when it was presented very briefly, whereas it was clearly visible when presented slightly longer. Although a prime could not be perceived it was still processed beyond the visual system, as evidenced by faster response times (RTs) and fewer errors when the prime and target were congruent than when they were incongruent, referred to here as the *correspondence effect* [12]. Interestingly, the conscious experience of response conflict on trial *n*–1 (previous trial) influenced cognitive control mechanisms on trial *n* (current trial), in such a way that the correspondence effect on trial *n* was smaller when trials were preceded by an incongruent trial compared to a congruent trial; here referred to as *conflict adaptation*.

These results are generally interpreted by assuming that, following the detection of conflict, PFC-driven cognitive control processes resolve conflict and increase future performance by increasing top-down control over sensory processes [15], [16], [17]. However, the occurrence of specific stimulus/response repetitions might also explain some variance in conflict tasks [18], [19], [20]. Crucially, in Kunde's experiment [12], whereas conflict adaptation was clearly present after the conscious experience of conflict, it was absent when conflict-inducing stimuli were experienced unconsciously, which suggests that conscious information processing is necessary for trial-by-trial regulatory changes in cognitive control. A lack of conflict adaptation after unconscious conflict has also been observed by others using similar paradigms [4], [21].

However, recently, we [22] and others [23] have shown that response errors that remained unaware can induce post-error slowing mechanisms in the subsequent trial, suggesting that unconscious information can influence cognitive processes for relatively long periods of time (in these cases at least ∼2 seconds). Interestingly, in EEG, we have also shown that unconscious errors triggered increased oscillatory phase synchrony between prefrontal and visual electrodes leading up to the next trial. Granger causality analysis suggested that these interactions were driven mainly by the prefrontal cortex. These findings suggest that, after unconscious errors, the PFC initiates a top-down biasing mechanisms that “instructs” visual cortex to improve sensory performance (to overcome future errors).

Here we test whether conflict adaptation can also be triggered unconsciously. In Kunde's experiment [12] a warning sign (a click sound) was presented before the presentation of each prime-target pair, which predicted the upcoming stimulus that was always presented 750 ms later. We reasoned that it might have been possible that participants released their attention after each trial and waited for the warning sign to reinstate their attentional focus. Therefore, the weak neural traces elicited by masked primes might have “died out” before the appearance of the next trial. Indeed, it has been shown that top-down attention facilitates the processing of unconscious information on the current trial [24], [25]. Furthermore, it has been proposed recently that top-down attentional processes might improve the ability to maintain the otherwise fleeting form of information carried by the unconscious stimulus and that when attention is released the ability to use this information disappears [22], [26]. Here, we slightly modified the task design of Kunde [12] in such a way that the presentation of a prime-target pair was not preceded by a warning sign. Additionally, we decreased the inter-trial interval in the present experiment, now ranging from ∼1200 to ∼1500 ms instead of ranging from 2050 to 2350 ms (and we did not include a neutral condition). The combination of these factors ensured that participants had to continue focusing on the location of the imminent stimulus during the (short) inter-trial interval for fast and accurate performance. Using this version of the masked priming task, we demonstrate trial-by-trial conflict adaptation effects after conscious as well as unconscious conflict.

## Materials and Methods

### Ethics statement

All procedures were executed in compliance with relevant laws and institutional guidelines and were approved by the ethics committee of the Psychology department of the University of Amsterdam. Subjects gave written informed consent before experimentation.

### Participants

Fifty-eight volunteers participated in a battery of tests (2 h) including the one described in the current paper (½h) for course credits or financial compensation. Non-overlapping results from this dataset have been published elsewhere [27].

### Design

**Figure 1:**
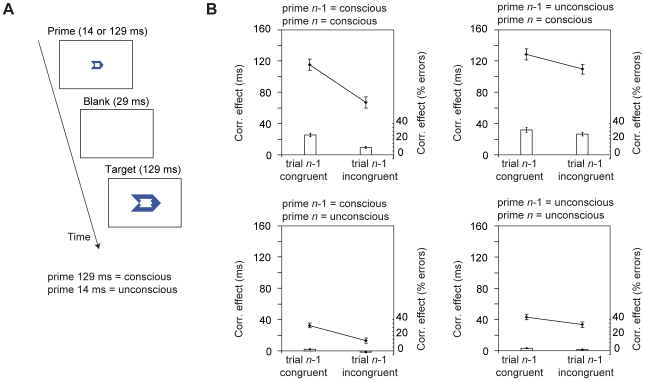
Experimental design and conflict adaptation results. A) Stimuli and trial timing. B) Conflict adaptation results. Correspondence effects in trial *n* for RTs (mean RT on incongruent trials–mean RT on congruent trials) and error rates (mean percentage of errors on incongruent trials–mean percentage of errors on congruent trials) as a function of prime-target correspondence in trial *n*-1 (congruent vs. incongruent), masking strength in trial *n* (weak vs. strong masking) and masking strength in trial *n*-1. Conflict adaptation was significant for all possible combinations of masking strength in trial *n* and masking strength in trial *n*-1.

Stimuli were presented against a white background at the centre of a 17-inch VGA monitor (frequency 70 Hz.), which was viewed from a distance of approximately 90 cm (each cm subtended a visual angle of 0.64°). A blue prime arrow (width 0.96°, height 0.64°) was presented for 14 ms or 129 ms, followed by a blank interval (29 ms), and then by a target arrow (129 ms, width 2.20°, height 1.47°) that instructed participants to respond as quickly and accurately as possible to its direction (Fig. 1a). Participants were instructed to ignore the prime, which was a smaller version of the target and fitted within the contour of the target. By manipulating prime duration, the prime was either visible (*weakly masked condition*), or its visibility was sharply reduced (*strongly masked condition*). Thus, four conditions were created: 1) weakly masked incongruent trials, 2) weakly masked congruent trials, 3) strongly masked incongruent trials, and 4) strongly masked congruent trials. Participants performed four blocks, each containing 160 trials (40 per condition, all 160 trials were shuffled randomly and subsequently drawn sequentially from this array of trials). Total trial duration was 1400, 1500, 1600 or 1700 ms (equal frequency). Before the experiment participants practiced the task briefly (32 trials). Performance feedback was presented after each block (mean RT, SD and percentage correct on congruent/incongruent trials).

After the main task, participants performed a two-alternative forced-choice discrimination task on the primes (80 trials; 20 trials of each condition). Stimulus and trial timing was exactly the same as in the main task, except that a pair of choices was presented left and right of fixation after each trial. Participants were asked to determine as accurately as possible whether a left-pointing or right-pointing prime was presented in the preceding trial. Before administrating this task, participants were told that left and right pointing primes were presented equally frequently and were instructed to consider this in giving their response. Accuracy was important in this task, not the speed of responding. Upon responding a new trial started.

### Data analysis

RTs <100 and >1000 were excluded from the analysis. Mean RTs on correct trials and square rooted accuracy rates were entered into an ANOVA with within-subjects' variables of prime–target correspondence in trial *n* (congruent vs. incongruent), masking strength in trial *n* (weakly masked vs. strongly masked), prime–target correspondence in trial *n*-1 and masking strength in trial *n*-1 [see 12]. All trials following errors were excluded from the analyses. Detection performance (percentage correct) was tested for significance for each individual participant using a binominal test evaluated at an alpha level of 0.05.

## Results

### Prime discrimination

The two-alternative forced-choice discrimination task administrated after the main task revealed that 42 out of 58 (72%) participants were unable to perceive the strongly masked primes, as evidenced by chance-level performance (binominal test). Because we cannot ascertain that the other 16 participants were truly unable to perceive the strongly masked primes consciously, they were excluded from further analyses. For the remaining 42 participants the percentage correct for weakly masked primes was 91.8% (SD = 12.6) vs. 54.8% (SD = 5.7) for strongly masked primes. Although prime discrimination was close to 50% when strongly masked, on a group level, it was significantly above chance-level (*t*(41)  = 5.46, *p*<0.001).

### Conflict adaptation reflected in RTs

Mean RTs and error rates of all factorial combinations of the included variables are presented in Table 1. Overall, participants responded slower to incongruent than to congruent trials (main effect of prime-target correspondence in trial *n*, *F*(1,41)  = 355.3; *p*<0.001) and also slower after experiencing conflict in the previous trial (main effect of prime-target correspondence trial *n*-1, *F*(1,41)  = 216.8; *p*<0.001). Furthermore, participants responded slower after a conscious prime in trial *n*-1 (main effect of masking strength in trial *n*-1, *F*(1,41)  = 157.1; *p*<0.001) and faster on trials with a conscious prime in trial *n* (main effect of masking strength in trial *n*, *F*(1,41)  = 116.8; *p*<0.001).

**Table 1:** Mean response times (in ms) and error rates (in percentages) as a function of masking strength in trial *n* and trial *n*-1 and prime-target correspondence in trial *n* and trial *n*-1.

	Trial *n*							
	Unconscious				Conscious			
	Incongruent		Congruent		Incongruent		Congruent	
Trial *n*-1	RT	ER	RT	ER	RT	ER	RT	ER
Unconscious								
Incongruent	488	4.0	454	2.6	506	28.3	397	1.9
Congruent	489	6.1	446	3.0	516	33.8	387	2.1
Conscious								
Incongruent	517	1.6	504	3.0	512	12.5	445	3.0
Congruent	496	4.3	464	1.9	510	27.4	395	2.2

*Note* - RT, response times; ER, error rate; unconscious, strongly masked primes; conscious, weakly masked primes.

As expected, the size of the correspondence effect in trial *n* (mean RT on incongruent trials–mean RT on congruent trials) was smaller when trials were preceded by an incongruent trial than when trials were preceded by a congruent trial (interaction prime-target correspondence in trial *n* and prime-target correspondence trial *n*-1, *F*(1,41)  = 78.8; *p*<0.001). This demonstrates conflict adaptation irrespective of prime awareness. Crucially, follow-up analyses revealed significant conflict adaptation effects for all possible combinations of masking strength in trial *n*-1 and masking strength in trial *n* (all *F*s>7.8; all *p*s<0.009, see Figure 1b). This indicates that conscious, but also unconscious incongruent primes in trial *n*-1 trigger conflict adaptation effects in trial *n* (irrespective of whether trial *n* contains a conscious or unconscious prime). However, conflict adaptation was stronger when trials were preceded by a weakly masked prime (conscious) than by a strongly masked prime (unconscious), as reflected in a 3-way interaction between prime-target correspondence in trial *n*, prime-target correspondence trial *n*-1 and masking strength in trial *n*-1 (*F*(1,41)  = 17.1; *p*<0.001). Finally, we observed a 4-way interaction between prime-target correspondence in trial *n*, prime-target correspondence trial *n*-1, masking strength in trial *n*-1 and masking strength in trial *n* (*F*(1,41)  = 6.0; *p* = 0.019), indicating that conflict adaptation was largest when conscious primes in trial *n*-1 were followed by conscious primes in trial *n*.

### Conflict adaptation reflected in error rates

The error analyses mirrored the RT analyses reported above. Participants made more errors on incongruent than on congruent trials (main effect of prime-target correspondence in trial *n*, *F*(1,41)  = 140.8; *p*<0.001). Furthermore, participants made more errors when trial *n*-1 contained an unconscious prime (main effect of masking strength in trial *n*-1, *F*(1,41)  = 46.4; *p*<0.001) and more errors when trial *n* contained a conscious prime (main effect of masking strength in trial *n*, *F*(1,41)  = 133.3; *p*<0.001). They made less errors after experiencing conflict in the previous trial (main effect of prime-target correspondence in trial *n*-1, *F*(1,41)  = 61.5; *p*<0.001).

Again, the size of the correspondence effect in trial *n* (mean error rate on incongruent trials–mean error rate on congruent trials) was smaller when trials were preceded by an incongruent trial than when trials were preceded by a congruent trial (interaction prime-target correspondence in trial *n* and prime-target correspondence in trial *n*-1, *F*(1,41)  = 58.9; *p*<0.001). Follow-up analyses revealed significant conflict adaptation effects for all possible combinations of masking strength in trial *n*-1 and masking strength in trial *n* (all *F*s >4.4; all *p*s <0.041, see Figure 1b). Just as observed in RTs, conflict adaptation was stronger when trials were preceded by a weakly masked prime (conscious) than by a strongly masked prime (unconscious), as reflected in a 3-way interaction between prime-target correspondence in trial *n*, prime-target correspondence trial *n*-1 and masking strength in trial *n*-1 (*F*(1,41)  = 9.6; *p* = 0.004). Finally, conflict adaptation was largest when conscious primes in trial *n*-1 were followed by conscious primes in trial *n* as reflected in a 4-way interaction between prime-target correspondence in trial *n*, prime-target correspondence in trial *n*-1, masking strength in trial *n*-1 and masking strength in trial *n* (*F*(1,41)  = 7.5; *p* = 0.009).

### Conflict adaptation and stimulus/response repetitions

**Figure 2:**
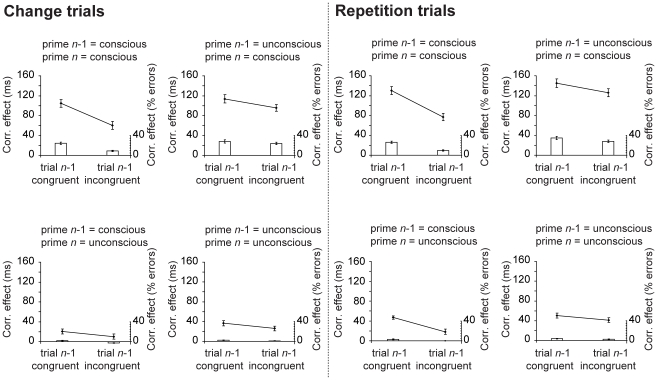
Conflict adaptation results for repeat and change trials separately. Correspondence effects in trial *n* for RTs (mean RT on incongruent trials–mean RT on congruent trials) and error rates (mean percentage of errors on incongruent trials–mean percentage of errors on congruent trials) as a function of prime-target correspondence in trial *n*-1 (congruent vs. incongruent), masking strength in trial *n*-1 (weak vs. strong masking), and masking strength in trial *n*. Correspondence effects are reported for a dataset containing trials without stimulus/response repetitions (*change trials*) and trials with stimulus/response repetitions only (*repetition trials*).

It is controversial whether these conflict adaptation effects are truly due to regulatory changes in cognitive control or whether they reflect mere lower-level priming effects [19], [28]. It has been argued that, especially in the Eriksen Flanker task, conflict adaptation effects might be mediated by particularly fast responses on trials preceded by trials with the same stimulus/response contingencies, especially evident for congruent trials following congruent trials and incongruent trials following incongruent trials with the same response. To test this issue, we re-analyzed the data in such a way that in one subset of the data all trials with direct stimulus/response repetitions were excluded (*change trials*), whereas in a second subset *only* trials with direct stimulus/response repetitions were included (*repetition trials*). The correspondence effect for RTs and errors as a function of prime-target correspondence in trial *n*-1, masking strength in trial *n*-1 and masking strength in trial *n* and dataset (repeat versus change) is depicted in figure 2. The conflict adaptation results were highly similar across change and repetition trials for RTs as well as error rates, as evidenced by the absence of significant 3-way interactions between prime-target correspondence in trial *n*, prime-target correspondence in trial *n*-1 and dataset for RTs (*F*(1,41)  = 2.6; *p* = 0.11) as well as error rate (*F*(1,41)  = 0.21; *p* = 0.65). Although, two-choice experiments do not allow a full-proof test against low-level priming effects these additional analyses as well recent other studies [e.g. 29] suggest that conflict adaptation cannot be fully explained by low-level priming effects.

### Prime visibility and adaptive control

To rule out the possibility that conflict adaptation triggered by strongly masked primes in trial *n*-1 was due to incidental visibility of the primes, we performed several additional analyses. First, we correlated the conflict adaptation effect for all trials with an unconscious prime in trial *n*-1 with detection performance (percentage correct on strongly masked trials) across participants to check whether both measures were related. Conflict adaptation (RTs or error rates) did not correlate with detection performance (all *p*s >0.72). Additionally, we selected the 50% (21 participants) worst detection performers (mean percentage correct = 50.1%) and tested conflict adaptation across these “poor detectors”. For this group, conflict adaptation effects were highly similar compared to the whole group. Crucially, conflict adaptation for trials preceded by strongly masked (unconscious) primes were still significant for RTs (prime *n* conscious: *F*(1,20)  = 5.5, *p* = 0.030; prime *n* unconscious: *F*(1,20)  = 4.5, *p* = 0.047) and error rates (prime *n* conscious: *F*(1,20)  = 5.4, *p* = 0.031; prime *n* unconscious: *F*(1,20)  = 3.3, *p* = 0.085). Also, conflict adaptation after conscious (weakly masked) primes remained significant for RTs and error rates irrespective of masking strength in trial *n* (all *p*s<0.028). These results further suggest that conflict adaptation effects (observed in trial *n*) can be induced by strongly masked primes (in trial *n*-1) that cannot be perceived consciously.

## Discussion

We report that unconsciously induced conflict can elicit conflict adaptation in the next trial. In a masked priming experiment, we focused on behavioral adaptations following conflict resulting from incongruent trials compared to behavioral adaptations after trials on which no conflict was experienced (congruent trials). Conflicting response tendencies were elicited either consciously (weakly masked primes) or unconsciously (strongly masked primes). We replicated the standard conflict adaptation effect for conscious conflict; the correspondence effect in trial *n* was sharply reduced after incongruent compared to congruent trials in trial *n*-1 (for response times as well as error rates). Crucially, conflict adaptation was also present after unconsciously induced conflict. These findings suggest that participants engender a more cautious response strategy and increase cognitive control after the experience of conscious, but also unconscious conflict-inducing stimuli.

Generally, conflict adaptation is interpreted by assuming that, following conflict, cognitive control processes subserved by the PFC resolve conflict and increase future performance by increasing top-down control over perceptual processes [15], [16], [17]. However, it has also been argued that correspondence effects can be explained fully by low-level repetition priming effects [18], [19]. Although repetition priming has been shown to explain variance on some occasions [19], [20], the present as well as several previous results could not be explained by simple stimulus/response repetitions across trials [e.g. 16,29,30].

Interestingly, using a similar design, Kunde [12] did not observe conflict adaptation after unconscious conflict [see also 4], [21], [29]. The crucial difference between his design and ours is the omission of a warning sign before stimulus presentation along with shorter trial durations in the present experiment. It might be that these two manipulations ensured that participants remained their attentional focus in between trials, instead of releasing their attention up until the presentation of the warning sign. Since it has been shown recently that (spatial and temporal) attention can be directed towards unconscious stimuli and that doing so increases the impact of these stimuli on subsequent behavior [24], [25], we hypothesize that, in line with others [26], such relatively long-term effects of unconscious information might be due to top-down attentional facilitation of the weak neural traces elicited by the unconscious primes. Future work is required to test this hypothesis more systematically.

Although speculative since we did not obtain any neural measures here, recent studies did observe relatively long-lasting neural activations elicited by masked (unconscious) words in a masked priming paradigm, up to approximately one second [26], [31]. Even longer effects of unconscious priming (up to several minutes) have been reported, for example in “mere exposure” paradigms [32], [33]. In combination, these results suggests that unconscious stimuli can influence cognitive processes for longer periods of time than previously thought, which has direct implications for theoretical models that propose a rapid decay of unconscious neural traces [34], [35], [36]. Future studies are necessary to further specify the temporal limitations of unconscious information processing, for example by systematically varying the inter-trial interval in a masked priming experiment.

In sum, we show that unconsciously experienced conflict-inducing stimuli can trigger conflict adaptation (under specific conditions). These results add to the growing body of literature suggesting that unconscious information can influence high-level (prefrontal) cognitive control functions, such as inhibitory control [9], [10], task switching [8], error correction [22], [23] and conflict adaptation (present study). These results further elucidate and expand the potential influence of unconscious information on our direct, but also future decisions.
